# A bacterial chloroform reductive dehalogenase: purification and biochemical characterization

**DOI:** 10.1111/1751-7915.12745

**Published:** 2017-06-20

**Authors:** Bat‐Erdene Jugder, Susanne Bohl, Helene Lebhar, Robert D. Healey, Mike Manefield, Christopher P. Marquis, Matthew Lee

**Affiliations:** ^1^ School of Biotechnology and Biomolecular Sciences University of New South Wales Sydney NSW 2052 Australia; ^2^ Department of Biotechnology Mannheim University of Applied Sciences 68163 Mannheim Germany; ^3^ School of Chemistry University of New South Wales Sydney NSW 2052 Australia

## Abstract

We report herein the purification of a chloroform (CF)‐reducing enzyme, TmrA, from the membrane fraction of a strict anaerobe *Dehalobacter* sp. strain UNSWDHB to apparent homogeneity with an approximate 23‐fold increase in relative purity compared to crude lysate. The membrane fraction obtained by ultracentrifugation was solubilized in Triton X‐100 in the presence of glycerol, followed by purification by anion exchange chromatography. The molecular mass of the purified TmrA was determined to be 44.5 kDa by SDS‐PAGE and MALDI‐TOF/TOF. The purified dehalogenase reductively dechlorinated CF to dichloromethane *in vitro* with reduced methyl viologen as the electron donor at a specific activity of (1.27 ± 0.04) × 10^3^ units mg protein^−1^. The optimum temperature and pH for the activity were 45°C and 7.2, respectively. The UV‐visible spectrometric analysis indicated the presence of a corrinoid and two [4Fe‐4S] clusters, predicted from the amino acid sequence. This is the first report of the production, purification and biochemical characterization of a CF reductive dehalogenase.

## Introduction

Environmental pollution with organohalides mainly caused by unregulated use and discharge from industrial sites is a serious public health problem due to their recalcitrance and hazardous impact on all ecosystems. Organohalide‐respiring bacteria (ORB) and their reductive dehalogenase (RDase; EC 1.97.1.8) enzymes represent promising bioremediation solutions to these pollutants, as organohalides are used as terminal electron acceptors for growth of ORB. During the reductive dehalogenation reaction catalysed by the RDase, one halogen atom is replaced by one hydrogen atom, yielding a lesser halogenated product which is in many cases less toxic and less recalcitrant to further dehalogenation. RDases are membrane‐associated redox metalloproteins with a corrinoid and two iron‐sulphur clusters as cofactors (Holliger *et al*., 1998; Jugder *et al*., 2015). Several RDases have been studied from obligate (such as, *Dehalobacter* and *Dehalococcoides* strains) and non‐obligate ORB (such as, *Desulfitobacterium* and *Sulfurospirillum* strains) (Jugder *et al*., 2015, 2016a). Most RDases derived from *Dehalococcoides* members, with the exception of CbrA from *D. mccartyi* CBDB1, were reported to dechlorinate chlorinated ethenes (Magnuson *et al*., 1998, 2000; Muller *et al*., 2004; Jugder *et al*., 2016a), whereas *Dehalobacter* RDases exhibited dechlorination activity towards chlorinated methanes, ethanes and ethenes (Schumacher *et al*., 1997; Tang and Edwards, 2013; Jugder *et al*., 2016a). The RDases reported from *Desulfitobacterium* strains mostly have substrate specificity for chlorinated ethanes and ethenes as well as *ortho*‐chlorophenols (van de Pas *et al*., 1999, 2001; Boyer *et al*., 2003; Thibodeau *et al*., 2004; Bisaillon *et al*., 2010).

Trichloromethane, commonly referred to by its trivial name chloroform (CF), is a hydrophobic (partition coefficient LogP_ow_ of 1.97 at 20°C) and volatile (vapour pressure of 197 mm Hg at 25°C) organohalide used as an industrial solvent for organic materials and as a chemical intermediate for the production of PTFE and the refrigerant mono‐chloro‐difluoro‐methane (HCFC‐22) (Verschueren, 1985; Jugder *et al*., 2016a). The low water solubility (< 8 g l^−1^ at 25°C), high density (ρ = 1.48 g cm^−3^) and long half‐life (3400 years at pH 7) contribute to its recalcitrance (Mabey and Mill, 1978; Mackay *et al*., 1980). The US‐EPA has classified CF as a probable human carcinogen (ASTDR, 2015) and it also causes cardiac, renal and hepatic toxicity (Larson *et al*., 1993; el‐Shenawy and Abdel‐Rahman, 1993). *Dehalobacter* sp. strain CF and *Desulfitobacterium* sp. strain PR have been reported to respire CF and their respective CF reductive dehalogenases (CfrA and CtrA respectively) were identified (Tang and Edwards, 2013; Ding *et al*., 2014).

*Dehalobacter* sp. strain UNSWDHB (*Dhb* sp. UNSWDHB) is an obligate ORB, which can dechlorinate CF to dichloromethane (DCM) (Wong *et al*., 2016). The bacteria can also utilize 1,1,1‐trichloroethane (TCA), 1,1,2‐TCA and 1,1‐dichloroethane (DCA) as electron acceptors. Its genome harbours 20 putative RDase homology (*rdh*) genes, of which only one (TmrA, gi736352001) was reported to be responsible for CF dechlorination and is expressed in response to CF addition (Deshpande *et al*., 2013; Jugder *et al*., 2016b; Wong *et al*., 2016). The maximum likelihood based phylogenetic analysis on the amino acid sequence of the characterized RDases revealed that TmrA shares high sequence homology with both CtrA and CfrA (Jugder *et al*., 2016a). The close homologies of TmrB (membrane anchor protein) and TmrC (putative transcriptional regulator) are also found as a similar genetic arrangement in *Dehalobacter* sp. CF (Table S1). Preliminary results for its CF reductase activity were reported in the crude cell extracts (Wong *et al*., 2016). However, isolation of the CF reductive dehalogenase has never been reported and there are no other reports of purified CF‐reducing dehalogenases, to our knowledge. Here, we describe the purification and biochemical characterization of TmrA from the UNSWDHB strain.

## Results

### Production of chloroform reductive dehalogenase, TmrA

To obtain sufficient biomass for protein purification, *Dhb* sp. UNSWDHB cells were cultivated with periodic additions of CF (approximately up to 0.4 mM day^−1^) and supplementation with hydrogen and carbon source (acetate) when necessary, for five months until harvesting. During this period the cultures in 2‐l bottles were closely monitored for their CF consumption and DCM production activity by gas chromatography (GC). The total amount of CF respired was approximately 60 mmol over the 5 month period. During the final days prior to harvest, CF dechlorination rate was 6.5 × 10^‐11^ mol cell^−1^ day^−1^ and the cell density was (9.8 ± 4.3) × 10^7^ cells ml^−1^. Before harvesting, we examined the expression of *tmrA* gene, as it was previously shown that the same gene is expressed upon CF respiration (Jugder *et al*., 2016b). To confirm the induction of *tmrA* at the gene level, transcription of *tmrA* gene was investigated. Qualitative gene‐expression analyses by RT–PCR demonstrated that the *tmrA* amplified from the cDNA synthesized from the cells actively respiring with CF was highly expressed, as the band intensities appear to reflect on the template abundancy (Fig. S1). The amplicon obtained with the tmrA_F and tmrA_R primers was purified and sequenced. A nucleotide sequence of about 190 bp was obtained for the PCR product, showing a 100% sequence identity to the *tmrA* gene of *Dhb* sp. UNSWDHB.

### Purification of chloroform reductive dehalogenase, TmrA

To purify TmrA, the cells grown with acetate, H_2_ and CF were anoxically harvested and disrupted mechanically. CF‐reductive dehalogenating activity was predominantly observed in the membrane fraction after ultracentrifugation of cell lysate. TmrA was solubilized from the membrane in the presence of 1% Triton X‐100 and purified 23‐fold by anion exchange chromatography using Mono Q HR as stationary phase (Table 1). The eluted fractions were tested instantly for dechlorination activity and CF‐reductase activity was recovered at approximately 200 mM NaCl as a single peak. The enzyme was purified to apparent homogeneity with an average specific activity of (1.27 ± 0.04)  × 10^3^ units per mg protein in the methyl viologen‐dependent assay (Table 1).

**Table 1:** Purification of CF reductive dehalogenase, TmrA

Purification	Total activity (U)^a^	Yield (%)	Total protein (mg)^b^	Specific activity (10^3^ U mg^−1^)	Purification factor
Cell extract	458	100	8.40	0.05	1
Membrane fraction	376	82	3.03	0.12	2
Solubilized membrane fraction	348	76	0.629	0.55	10
Mono Q HR	172	38	0.135	1.27	23

**a.** Amount (nmol) DCM produced per min with CF as substrate,

**b.** Total protein was determined with Pierce 660 nm protein assay using bovine serum albumin as a standard.

**Figure 1:**
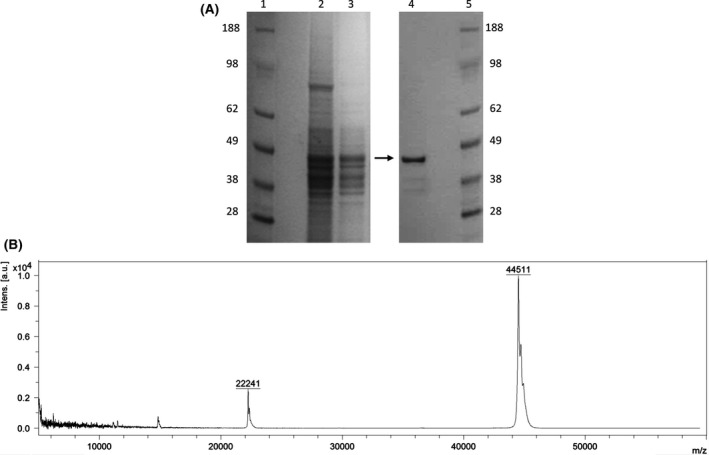
The purified TmrA. A. SDS‐PAGE with the purified chloroform reductive dehalogenase, TmrA, of *Dehalobacter* UNSWDHB in *lane 4*. Molecular size markers (SeeBlue Plus2 Prestained Standard; Thermo Fisher Scientific) are shown in *lane 1 and 5*. The crude cell lysate and solubilized membrane fraction are in *lane 2 and 3*. The arrow indicates the purified protein band. The gel was stained with GelCode Blue Stain Reagent (Thermo Fisher Scientific). B. MALDI‐TOF spectra of the TmrA at an intact mass of 44 511 Da.

**Figure 2:**
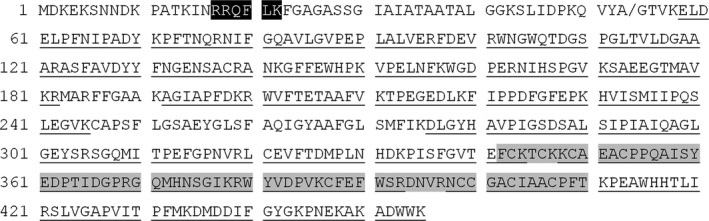
Deduced amino acid sequence of TmrA from strain UNSWDHB. The black and grey boxes indicate the TAT signal peptide and a [4Fe‐4S] double cluster binding domain (NCBI: pfam13484), respectively. Underlined sequences are peptides that were detected by mass spectrometry with MASCOT (coverage 77.3%, score of 2223). A slash indicates the predicted location of the cleavage of the peptide signal predicted by PRED‐TAT.

The MALDI‐TOF analysis of purified native TmrA revealed an intact mass detection of 44 511 Da, corresponding to the apparent molecular mass from the isolated band found in SDS‐PAGE (Fig. 1). After purification and SDS‐PAGE separation, the mass band at 45 kDa was excised from the polyacrylamide gel and sequenced by proteolytic degradation and LC‐MS/MS peptide identification. The sequence of a full‐length TmrA contains a Tat‐signal peptide at its N‐terminus; however, in this analysis the N‐terminal detected peptide sequence started at amino acid number 58, suggesting N‐terminal cleavage prior to translocation to the periplasmic membrane. Using the PRED‐TAT program (Fig. 2) (Bagos *et al*., 2010) suggests that the most likely cleavage site is between residue 53 (Ala) and residue 54 (Gly). As such, the expected mass of the intact protein without the signal peptide is 44 522.8 Da (calculated using ProtParam‐ExPaSy), which very slightly differs to the mass detected by MALDI‐TOF.

### Biochemical properties of chloroform reductive dehalogenase

**Figure 3:**
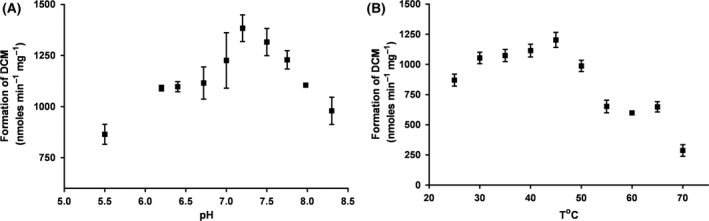
The effect of pH and temperature on the activity of the purified TmrA. A. The anaerobic reaction mixtures containing the purified TmrA (4 μg), 2 mM titanium (III) citrate and 2 mM methyl viologen in 100 mM Tris–HCl buffer at pH values of 5.5, 6.2, 6.4, 6.72, 7.0, 7.2, 7.5, 7.75, 7.98 and 8.3 were incubated at 30°C. B. The mixtures were incubated at temperatures between 25 and 70°C, with 5°C increments, at pH 7.5. The average values of triplicate assays are presented.

The pH dependency of TmrA activity was tested in the standard assay reaction mixtures (30°C) containing 4 μg of the purified enzyme, 2 mM titanium (III) citrate and 2 mM methyl viologen in 100 mM Tris–HCl buffer at various pH values (5.5–8.3). The pH optimum of TmrA enzyme was found to be 7.2 (Fig. 3A). The optimum temperature for dechlorinating activity was evaluated by testing activity between 25 and 70°C (pH 7.5), which resulted in 45°C being found to be the optimum value (Fig. 3B). A substantial decrease in enzyme activity was observed at temperatures higher than 55°C.

The substrate range of the purified TmrA is shown in Table 2. The highest specific activity was observed with CF, followed by 1,1,2‐TCA, 1,1,1‐TCA and 1,1‐DCA. The substrate specificity of the purified enzyme is in agreement with that reported for the *Dhb. sp* UNSWDHB cell extracts (Wong *et al*., 2016). This confirms that a single enzyme is responsible for the dechlorination of these organohalides by this bacterium under the experimental condition used.

**Table 2:** Substrate specificity profile of purified TmrA

Substrate	Product(s)	Mean specific activity (10^3^ nmol min^−1^ mg of protein^−1^)
Chloroform	Dichloromethane	1.27 ± 0.04
1,1,2‐trichloroethane	1,2‐dichloroethane, vinyl chloride	1.15 ± 0.01
1,1,1‐trichloroethane	1,1‐dichloroethane, chloroethane	0.12 ± 0.01
1,1‐dichloroethane	Chloroethane	0.03 ± 0.01

The mean values of duplicates are presented with standard deviations.

**Figure 4:**
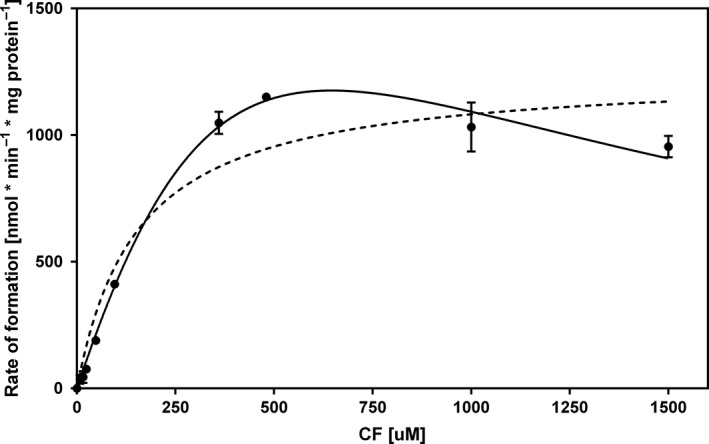
CF dechlorinating kinetics of purified TmrA. Each point represents the initial rate of dechlorination determined by the concentration of DCM formed after 2 h incubation. Curves are nonlinear regression fitted to the Substrate Inhibition model (solid line) and the Michaelis–Menten model (dashed line), resulting from duplicates using GraphPad Prism v. 6.07. Error bars represent standard deviation.

According to the Michaelis–Menten model, the apparent *K*_m_ value of the purified enzyme for CF was 154 ± 41 μM and the apparent *V*_max_ value was (1.25 ± 0.09) × 10^3^ nmol min^−1^ mg of protein^−1^ for CF at a methyl viologen concentration of 2 mM. The highest initial activity of (1.28 ± 0.04)  × 10^3^ nmol min^−1^ mg of protein^−1^ was measured at 480 μM of CF. A decrease in the dechlorination rate was observed when substrate concentrations exceeding this value were used. The reaction kinetics modelled to include substrate inhibition (with an inhibition constant (*K*_i_) of 354 μM) fitted experimental data better (*R* ^2^ = 0.998) than the Michaelis–Menten model (*R* ^2^ = 0.971) over the whole substrate concentration range examined (Fig. 4).

**Figure 5:**
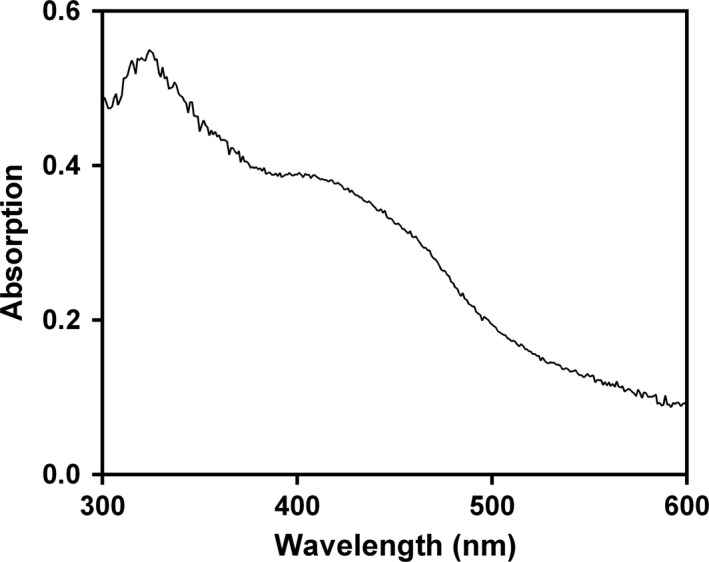
UV‐visible absorption spectra of the purified TmrA. The purified enzyme (0.05 mg ml^−1^) exhibits features consistent with inclusion of both [4Fe‐4S] clusters (∼420 nm) and cob(II)alamin (310 nm and 450–475 nm) as cofactors.

The native protein presents a broad absorption maximum between 400 and 500 nm and a shoulder at 310 nm (Fig. 5). The shape and location of these bands are typical of iron–sulphur proteins, including previously isolated reductive dehalogenases (Neumann *et al*., 1996; Schumacher *et al*., 1997; Christiansen *et al*., 1998). This is further substantiated by the absorption maximum at around 420 nm, which is assigned to the [4Fe‐4S] clusters. The absorbance in the 450–475 nm region as well as the shoulder at 310 nm could indicate the presence of a cobalamin in the 2+ oxidation state (Neumann *et al*., 1996; Christiansen *et al*., 1998; Miles *et al*., 2015). Moreover, the lack of peaks at 385 nm or at 360 nm, characteristic for cob(I)alamin or cob(III)alamin, respectively, suggests the presence of cobal(II)amin in the dehalogenase, which is indeed a characteristic of many corrinoid proteins (Banerjee, 1999).

## Discussion

*Dehalobacter* sp. strain UNSWDHB is able to use CF as a terminal electron acceptor for growth. It has previously been demonstrated that the enzyme directly responsible for reductive dechlorination of CF is the membrane‐associated TmrA, which is specifically overproduced in response to CF (Jugder *et al*., 2016b; Wong *et al*., 2016). In this study, we for the first time report the production, purification and biochemical characterization of a chloroform‐reducing enzyme. The TmrA dehalogenase was purified to near homogeneity from *Dehalobacter*. sp strain UNSWDHB cells in the presence of CF. Most of the dechlorinating activity was found in the membrane fraction, as observed in most RDases purified previously, due to the translocation of the enzymes to the periplasmic membrane via TAT signal pathway (Jugder *et al*., 2015, 2016a). A simple purification protocol was developed, involving membrane fractionation and solubilization in the presence of Triton X‐100 followed by a step gradient elution in anion exchange chromatography. The estimated specific activity of 1.27 × 10^3^ nmol min^−1^ mg protein^−1^ is in the middle range of other native RDases reported previously (Jugder *et al*., 2016a), although it should be noted that there were differences in the activity assay methods employed among the studies (e.g., gas chromatography versus spectrophotometry, measurement of substrate dechlorination versus product formation, reaction conditions).

The purification of native RDases from wild‐type ORB, especially from obligate respirers like *Dehalobacter*, is challenging due to the difficulty in obtaining sufficient biomass, oxygen intolerance of ORB and low water solubility of organohalides. Nevertheless, several RDases have been isolated from facultative and obligate ORB; some of them followed similar strategies used in this study. The PCE‐degrading PceA from *Dehalobacter restrictus* was purified using membrane solubilization with Triton X‐100, ultracentrifugation and anion exchanger (Schumacher *et al*., 1997). Van de Pas and co‐workers purified the 3‐chloro‐4‐hydroxyphenylacetate‐dechlorinating CprA from *Desulfitobacterium sp*. sp. PCE‐1 and the ortho‐chlorophenol‐degrading CprA from *Desulfitobacterium dehalogenans,* the PCE‐degrading PceA from *Desulfitobacterium. *sp. PCE‐1 and from *D. hafniense* TCE‐1 (van de Pas *et al*., 1999, 2001) using the same solubilization technique, followed by consecutive anion exchange steps. Other native RDases, such as the PCE, TCE, VC‐degrading RDases from *Dehalococcoides* and *Sulfurospirillum* members, have been purified in a functional form employing similar approaches (Magnuson *et al*., 1998, 2000; Muller *et al*., 2004).

The molecular mass of the enzyme detected by MALDI‐TOF analysis (44 511 Da) was in agreement with the 45 kDa‐protein band on SDS‐PAGE. This is in the same range of molecular mass of biochemically characterized RDases to date (30–64 kDa) (Jugder *et al*., 2016a). Although the predicted mass of the full‐length protein devoid of the region containing the signal peptide (44 522 Da) differs by 11 Da, such difference can be attributed to the analytical techniques, as MALDI‐TOF detects the intact protein including post‐translational modifications, whereas LC‐MS/MS detects digested peptide fragments and post‐translational modifications were not included in the search. Previously, in our laboratory, TmrA was identified as putatively dimeric, with an approximate mass of 100 kDa using blue native polyacrylamide gel electrophoresis (BN‐PAGE) coupled with LC‐MS/MS (Wong *et al*., 2016). The purification of the native TmrA under reducing conditions may suggest dimer formation arises via intermolecular disulphide bonds.

The pH optimum at 7.2 (30°C) and temperature optimum at 45°C (pH 7.5) were in the range of values from previously reported similar enzymes (Boyer *et al*., 2003; Thibodeau *et al*., 2004; Bisaillon *et al*., 2010). It is worth mentioning that determined pH and temperature optima have to be regarded as solely local maxima. In case of process development, it would be justified determining the optimal parameters as pivoting function of both. The apparent *K*_m_ value of the purified enzyme for CF (149 μM) differs from the *K*_m_ value of the crude cell extract (30 μM), reported previously in our laboratory (Wong *et al*., 2016). The difference in *K*_m_ values between crude cell extract and the pure protein can be attributed to the surrounding environment of the protein (e.g., buffers, detergents, presence of other proteins and membrane components) that can affect the conformation of the protein. Higher concentrations of CF appear to inhibit the enzyme activity (*K*_i_ = 354 μM). Similar inhibition patterns caused by high substrate concentrations were observed with tetrachloroethene (PCE) and trichloroethene (TCE) dechlorinating enzymes (Neumann *et al*., 1996; Miller *et al*., 1998).

This study presents the first report on the isolation and biochemical characterization of a highly purified reductive dehalogenase enzyme that can catalyse the dechlorination of CF. Previous studies mainly reported multi‐step purification of RDases, whereas in this study the TmrA enzyme was purified to apparent homogeneity using membrane separation and solubilization followed by a single‐step liquid chromatographic purification. Kinetic parameters are reported for reduction in not only CF but three other substrates 1,1,1‐trichloroethane (TCA), 1,1,2‐TCA and 1,1 dichloroethane (DCA).

## Experimental procedures

### Growth, maintenance and harvest of cells

*Dhb* sp. UNSWDHB cells, previously isolated from a CF‐respiring enrichment culture (Lee *et al*., 2012), were grown at 30°C anaerobically in 2‐litre flasks containing defined mineral medium with hydrogen as the electron donor (Lee *et al*., 2012; Wong *et al*., 2016) and CF as the electron acceptor. The growth of the cells was monitored using cell counting under fluorescence microscopy. Glutaraldehyde and SYBR Green fluorescent stain (Thermo Fisher, USA) were used as the fixing and staining solution respectively. The stained cell suspensions were fixed onto a 1.5% agarose‐coated microscope slide and were imaged on a fluorescent microscope OLYMPUS BX51WI (Olympus‐Lifescience, USA). The medium and all buffers used were prepared using anaerobic techniques and contained 1 mM dithiothreitol (DTT). Working inside an anaerobic chamber Whitley DG250 (Don Whitley, West Yorkshire, UK) under a gas mixture containing 80% N_2_, 10% H_2_ and 10% CO_2_, 2 l of anaerobic cultures were transferred to airtight centrifuge tubes modified with PVC thread tape and harvested by centrifugation at 10 000 *g* for 45 min at 4°C. The cell pellets were washed in 40 ml of anaerobic 100 mM Tris buffer, pH 7.5, with 5% (w/w) glycerol, 1 mM DTT and 150 mM NaCl and subjected to another round of centrifugation under the same condition. The cell pellets were stored anoxically at −80°C until further use.

### Preparation of membrane fraction and purification of chloroform reductive dehalogenase

Approximately, 1 g of wet biomass was thawed and resuspended in anoxic 100 mM Tris buffer, pH 7.5, with 5% (w/w) glycerol, 1 mM DTT, 150 mM NaCl, cOmplete, EDTA‐free Protease inhibitor (Roche Applied Science, Penzberg, Germany) and DNase I (final concentration 20 μg ml^−1^). A ratio of cell wet weight to the resuspension buffer of 1:10 was used. The cell resuspensions were then allocated into 2 ml O‐ring capped FastPrep^®^ Lysing Matrix Tubes (MP Biomedicals, Solon, OH, USA) for cell disruption by bead beating at 50 Hz for 15 min using TissueLyser LT (QIAGEN, Hilden, Germany). Following a brief centrifugation to separate the beads, the crude cell lysate was transferred to a 12PA seal tube (Hitachi, Tokyo, Japan) and heat sealed using Tube Topper (Beckman, Palo Alto, CA, USA). The membrane fraction was separated at 140 000 *g* for 90 min at 4°C using CP100WX ultracentrifuge with rotor P90AT (Hitachi). Back in the anaerobic chamber, the supernatant was removed and the pellet was resuspended in 20 ml solubilization buffer (100 mM Tris, 10% (w/w) glycerol, 1 mM DTT and 1% (v/v) Triton X‐100, pH 8.0) and agitated for 1 h at 4°C for solubilizing the membrane fraction. After a second ultracentrifugation under the same condition, the supernatant containing the solubilized membrane fraction was immediately applied to a Mono Q HR (1 ml) column equilibrated with anaerobic buffer A (50 mM Tris, pH 8, 0.1% Triton X‐100, 10% glycerol and 1 mM DTT). Following washing with 20 column volumes (CVs) of buffer A, protein was eluted at a flowrate of 0.6 ml min^−1^ with a linear gradient of 20 CVs from 0 to 300 mM NaCl in buffer A. Fractions with chloroform‐reducing activity eluted in a peak at approximately 200 mM NaCl.

### Measurement of chlorinated methanes by gas chromatography

To monitor optimal growth of *Dhb* sp. UNSWDHB cells, the chlorinated methanes in the cultures (reduction of CF to DCM) were monitored by headspace analysis using an Agilent Technologies gas chromatograph equipped with a flame ionization detector (GC‐FID) and a GS‐GasPro capillary column (60 × 0.32 mm), as described elsewhere (Lee *et al*., 2012). Inlet and detector temperatures were set at 250°C, the oven temperature was programmed as follows: 1 min at 100°C followed by a gradient of 25°C min^−1^ increasing to 250°C where held for 5 min. The retention times of DCM and CF were 5.3 and 6 min, respectively, at a flowrate of 3 ml min^−1^.

### Dehalogenase activity assay

Standard enzyme assays were performed anoxically in the anaerobic chamber, as previously described with the following modifications (Grostern *et al*., 2009). Briefly, in 2 ml glass screw cap glass vials 1.5 ml of 100 mM Tris–HCl buffer (pH 7.5) containing 2 mM titanium (III) citrate and 2 mM methyl viologen was added. To each vial was then added 100 μl of crude protein extract or purified protein sample. The vials were then capped and CF with a final concentration of 0.5 mM was injected through the septum, leaving no headspace. The vials were incubated at 30°C inside the chamber for 5 h. After incubation, the enzymatic reaction was quenched by transferring 1 ml of each reaction mixture to a 10 ml headspace flask containing anhydrous sodium sulphate (0.5 g) and 1M sulphuric acid (1 ml). The headspace vials were then analysed by a Shimadzu 2010 GC‐FID equipped with a PAL Systems headspace auto‐sampler (PAL LHS2‐xt‐Shim) with GS‐Q capillary column (30 m × 0.320 mm) as stationary phase. Separation was carried out at 100°C for 1 min followed by 5 min ramping with 20°C min^−1^ to reach a final temperature of 250°C. Helium with a flowrate of 30 ml min^−1^ was used as the carrier gas. CF and DCM standards (0 mM, 0.5 mM, 1.0 mM, 1.5 mM and 2.0 mM) were prepared on the same day from 8 mM stock solutions in the same buffer and treated the same way as the samples. Enzyme specific activity was defined as the formation of 1 nmol of DCM per mg of protein per minute. As negative controls, heat‐deactivated crude protein extracts (prepared by incubating at 80°C for 15 min) as well as no CF mixtures were assayed in preliminary tests. Standard activity assays were performed in triplicate.

### Analytical methods

The total protein concentration was measured using a Pierce 660 nm protein assay kit (Thermo Fisher Scientific, Rockford, IL, USA). Proteins were separated on Bolt™ 4–12% Bis‐Tris Plus polyacrylamide precast gels (Thermo Fisher Scientific) with the SeeBlue Plus2 Prestained Standard (Thermo Fisher Scientific) as a molecular weight marker and stained with GelCode Blue Stain Reagent (Thermo Fisher Scientific). Protein bands on SDS‐PAGE were cut from the gel and digested in‐gel with trypsin as previously described (Hellman *et al*., 1995) for mass spectrometry analysis. The tryptic peptides were separated by nano‐LC using an Ultimate 3000 HPLC and autosampler system (Dionex, Amsterdam, Netherlands) using a micro C18 pre‐column (500 μm × 2 mm; Michrom Bioresources, Auburn, CA, USA). Mass spectra were recorded on an Orbitrap Velos (Thermo Electron, Germany) mass spectrometer equipped with a nanoelectrospray ionization source. Peak lists were generated using Mascot Daemon/extract_msn (Matrix Science, London, UK) using the default parameters and submitted to the database search program Mascot (version 2.2; Matrix Science). Mass spectra were searched against a custom database consisting of all proteins in the genome of *Dhb* sp. UNSWDHB.

The molecular mass of the purified TmrA was determined using MALDI‐TOF/TOF. The purified detergent solubilized TmrA protein solution was subjected to reverse‐phase C‐18 ZipTip clean up prior to analysis as per the manufacturer's instructions. Briefly, a C18 ZipTip was equilibrated with acetonitrile/water/trifluoroacetic acid (2/98/0.1%, v/v/v). 10 μL of detergent solubilized TmrA was aspirated over the ZipTip several times. The bound analyte was washed with equilibration solution and then eluted with a saturated solution of sinapic acid in acetonitrile/water/trifluoroacetic acid (80/20/0.1%, v/v/v). The eluted analyte was spotted directly onto a polished steel MALDI‐TOF target plate (Bruker Daltonics, Leipzig, Germany) and subject to MALDI‐TOF using a Bruker UltrafleXtreme MALDI‐TOF/TOF.

UV‐visible spectra of the purified dehalogenase were obtained with a Cary 60 UV‐Vis spectrophotometer. Absorbance of the dehalogenase sample in 50 mM Tris, pH 8, containing 0.1% Triton X‐100 and 10% glycerol was measured at between 300 and 600 nm in 1 cm quartz cuvettes.

The enzyme kinetic parameters, such as the apparent *K*_m_ and *V*_max_ values for CF, were determined under the standard assay conditions with a range of substrate concentration varying from 8 to 1500 μM. The parameters were calculated using GraphPad Prism v. 6.07, ‘Enzyme kinetics – Michaelis‐Menten’ module (GraphPad Software, Inc, La Jolla, CA, USA). Inhibition constant (*K*_i_) was determined by a nonlinear fit of experimental data to a substrate inhibition equation using the same software (‘Enzyme kinetics – Inhibition: Substrate inhibition’ module).

The optimum pH of the enzyme was determined by carrying out standard enzyme assays at different pH values between 5.5 and 8.3 in 100 mM Tris–HCl buffer at 30°C. The temperature dependence was tested by varying the incubation temperature between 25°C and 70, with 5°C increments, at pH 7.5. The average values of triplicates were used to create the graphs by GraphPad Prism v. 6.07.

The substrate range was tested on 1,1,2‐TCA, 1,1,1‐TCA and 1,1‐DCA under the standard activity assay conditions, with 1.5 mM of respective substrates.

### Semi‐quantitative reverse transcription RT‐PCR analyses

Total RNA for RT‐PCR from *Dhb* sp. UNSWDHB grown in the absence and presence of CF was isolated using RNeasy Mini Kit (Qiagen, Hilden, Germany) according to the manufacturer's instructions, including on‐column DNase digestion with the QIAGEN RNase‐Free DNase Set. The cDNA was synthesized from total RNA using the SuperScript VILO MasterMix kit (Life Technologies, Carlsbad, CA, USA), according to the manufacturer's protocol. Equivalent amounts of RNA (100 ng) from each sample were subjected to RT reaction. A No‐RT control was run with DEPC‐treated water instead of template RNA. The primers specifically designed for *tmrA* gene (NCBI GI number: 530294411), tmrA_F (5′‐TTTGCCCAGATTGGATATG‐3′) and tmrA_R (5′‐CTTCACAGAGTCTAACATTTG‐3′), were used in PCR reactions where annealing temperature of 57.6°C was applied. A no template control (NTC), replacing the template cDNA with DEPC‐treated water and a no‐RT were included as negative controls. For DNA amplification, 2× PCR Master Mix (Promega, USA) was used. The PCR products were separated via electrophoresis in a 1.8% (w/v) agarose gel and were subsequently cleaned up using DNA Clean & Concentrator‐5 kit (Zymo Research) with subsequent verification by sequencing by the method of Sanger [13] using the ABI 3730 Capillary Sequencer with BigDye™ Terminator Cycle Sequencing Ready Reaction kit v.3.1 (Applied Biosystems, Foster City, CA, USA).

## Conflict of interest

None declared.

## Supporting information


**Fig. S1.** Transcriptional profiling of the *tmrA* gene in *Dehalobacter* sp. UNSWDHB by qualitative RT–PCR.

**Table S1.** Genetic characteristics of chloroform reductive dehalogenase proteins of *Dehalobacter* sp. UNSWDHB.
