# Supplementary figures and images for: A bacterial chloroform reductive dehalogenase: purification and biochemical characterization
Source: Microb Biotechnol. 2017 Jun 20;10(6):1640–8. doi: 10.1111/1751-7915.12745 (PMC5658581; doi:10.1111/1751-7915.12745)

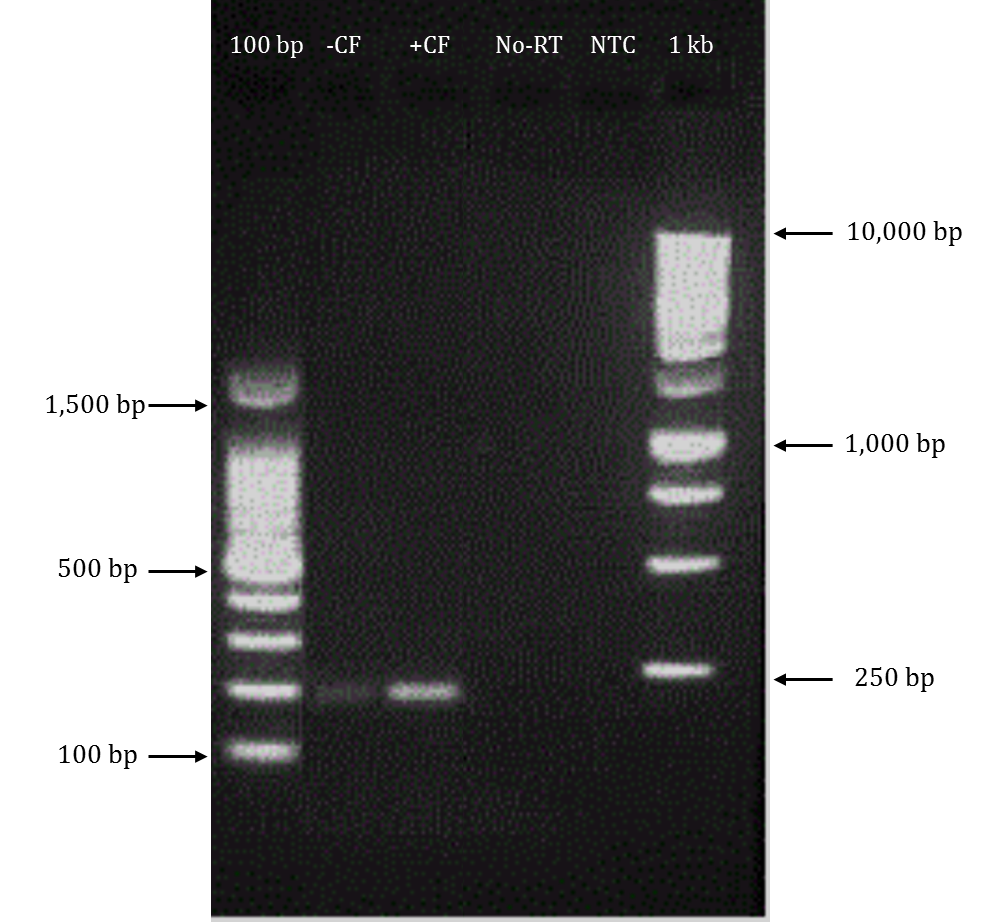

Supplement: Supplementary file 1 — Fig. S1. Transcriptional profiling of the tmrA gene in Dehalobacter sp. UNSWDHB by qualitative RT–PCR. [file MBT2-10-1640-s001.tif]
