# Supplementary material for: A bacterial chloroform reductive dehalogenase: purification and biochemical characterization
Source: Microb Biotechnol. 2017 Jun 20;10(6):1640–8. doi: 10.1111/1751-7915.12745 (PMC5658581; doi:10.1111/1751-7915.12745)
Supplement: Supplementary file 2 — Table S1. Genetic characteristics of chloroform reductive dehalogenase proteins of Dehalobacter sp. UNSWDHB. [file MBT2-10-1640-s002.docx]

**Supporting information**

**Fig. S1.** Transcriptional profiling of the *tmrA* gene in *Dehalobacter* sp. UNSWDHB by qualitative RT–PCR. The *tmrA* gene was amplified from cDNA obtained from the cells with and without CF (+CF and -CF).

**Table S1** Genetic characteristics of chloroform reductive dehalogenase proteins of *Dehalobacter* sp. UNSWDHB.

| **Protein name** | **Locus tag (NCBI) (UNSWDHB_RS#), protein length** | **Chromosomal location** | | **ORF Size (bp)** | **GC %** | **Closest homolog**  **Organism, locus, protein, identity** | **TMH** | **Fe-S binding domains** | **TAT signal** |
| --- | --- | --- | --- | --- | --- | --- | --- | --- | --- |
|  |  | **Start (bp)** | **Stop**  **(bp)** |  |  |  |  |  |  |
| TmrA (Chloroform reducing dehalogenase, catalytic subunit A) | 00295, 455 aa | 19936 | 21303 | 1368 | 44.0 | *Dehalobacter* sp. CF, DCF50_p1247, 456 aa, 95% | 1 | 4Fe-4S double cluster binding domain | RRQFLK |
| TmrB (Chloroform reducing dehalogenase, membrane anchor subunit B) | 00300, 105 aa | 21322 | 21639 | 318 | 40.2 | *Dehalobacter* sp. CF, DCF50_p1246, 105 aa, 99% | 3 | n/a | n/a |
| TmrC (Chloroform reducing dehalogenase transcriptional regulator) | 00305, 400 aa | 21830 | 23071 | 1242 | 37.3 | *Dehalobacter* sp. CF, DCF50_p1245, 448 aa, 94% | 6 | 4Fe-4S binding domain | n/a |

TMH, transmembrane helices are predicted by Philius transmembrane prediction server ([Reynolds et al., 2008](#_ENREF_26)).
